# Divergent Thinking in Early Childhood: Dimensions That Support Pre-Literacy Skills

**DOI:** 10.3390/jintelligence14080163

**Published:** 2026-08-01

**Authors:** Claudia Flores, Débora Areces, Celestino Rodríguez, Inés López-Manrique

**Affiliations:** 1 Department of Psychology, University of Oviedo, 33003 Oviedo, Spain; floresclaudia@uniovi.es (C.F.); rodriguezcelestino@uniovi.es (C.R.); 2 Department of Educational Sciences, University of Oviedo, 33003 Oviedo, Spain; lopezines@uniovi.es

**Keywords:** cognitive skills, pre-literacy skills, creative elaboration, preschool education, learning difficulties, assessment, explanatory power

## Abstract

Divergent thinking is a key educational skill associated with cognitive and pre-literacy abilities. However, the limited research conducted with preschool populations makes it difficult to draw firm conclusions and to inform curriculum changes at early ages. This study aimed to (1) examine sex and school grade differences in divergent thinking at this stage, and (2) analyze the explanatory power of creative variables for preliteracy skills in second-cycle preschool students. A total of 80 children aged 3 to 5 years (*M* = 4.44; *SD* = 0.57) participated in this study. They completed an ad hoc figurative drawing test based on widely used instruments created for older age groups. More specifically, this test assessed fluency, flexibility, originality, and elaboration (Cronbach’s alpha = 0.89). Additionally, the AEI-R battery was administered to evaluate cognitive and pre-literacy skills. Multivariate analyses revealed only limited sex-related differences in divergent thinking variables, while no statistically significant differences emerged by grade level. Furthermore, hierarchical regression analysis indicated that creative elaboration offered incremental explanatory power, contributing uniquely and significantly to the variance in pre-literacy skills. Overall, these findings suggest associations between specific dimensions of divergent thinking and cognitive and pre-literacy variables in early childhood.

## 1. Introduction

Children are active, curious, and capable beings with a variety of interests, skills, and knowledge. During early childhood, they actively explore their environment, guided by genuine curiosity and engaging in meaningful interactions that shape their understanding of the world (Đuranović et al., 2020; Hariawan et al., 2019). The period before the age of 6 is crucial for cognitive and socio-emotional development, as it lays the basis for future learning (Jurčević & Tot, 2020; Yildirim & Yilmaz, 2023). During this stage, fundamental skills emerge, such as intelligence (Józsa et al., 2022), pre-literacy skills (Xiao et al., 2023), and divergent thinking (Duval et al., 2022), the latter being recognized in preschool curriculum as an important competence to be fostered (García et al., 2016). Accordingly, understanding how divergent thinking relates to early cognitive and pre-literacy development may contribute to improving educational practices during the preschool years.

Divergent thinking, conceptualized as a complex multidimensional ability, is influenced by psychological, social, cultural, and material factors (Hernández-Jorge et al., 2022; W. Zhang et al., 2020). This capacity is associated with generating original ideas and solving open-ended problems (de Vries & Lubart, 2017; Goldschmidt, 2016). In pre-school, encouraging divergent thinking is essential as it promotes exploration and discovery, helping to maintain divergent thinking potential, which tends to diminish with age and schooling (Runco & Jaeger, 2012).

In addition, it is typically assessed through verbal and figurative indicators across four dimensions: fluency, flexibility, originality and elaboration (Guilford, 1967; Sternberg & Lubart, 1999). Fluency refers to the ability to generate many responses in a short time, flexibility to the ability to shift focus and consider multiple perspectives, originality to the creation of novel ideas, and elaboration to the detailed development of these ideas (Crenshaw & Miller, 2022; Hendrik et al., 2022; Handayani et al., 2021). Moreover, the expression of divergent thinking may vary according to the individual’s stage of development, from childhood to adolescence (Hong & Milgram, 2010). In the preschool stage, a divergent thinking potential characterized by subjectivity and egocentrism is manifested, which evolves to reach its full development in adolescence (Sawyer, 2012).

However, assessing divergent thinking in preschool children remains challenging. The lack of consensus regarding its nature and expression during early childhood has complicated its investigation in educational research (Nikkola et al., 2024). Although other aspects of child development have been extensively studied using validated instruments, research on divergent thinking in young children remains comparatively limited (Massy et al., 2025). Most studies have focused on primary and secondary education, often overlooking the specific developmental characteristics of the early years. Therefore, further research is needed to explore how divergent thinking manifests during early childhood and how it relates to cognitive and pre-literacy skills within educational settings.

The importance of addressing this issue is further emphasized by the widespread access to Preschool Education. According to a report by the National Institute of Statistics (INE) in Spain, in 2017, the schooling rate for children between the ages of 3 and 5 reached almost 100% (R. Zhang, 2023). With such a high enrolment rate, Preschool Education institutions play a crucial role in fostering children’s development. Consequently, Preschool curricula should align with children’s developmental needs and interests, promoting flexible, open, and meaningful learning experiences (Sylva et al., 2020).

In this regard, fostering divergent thinking during the preschool years may represent an important educational objective (Dere, 2019; Sau et al., 2020). However, one of the main challenges in this endeavor is the considerable interindividual variability observed during early childhood development, which complicates the assessment of divergent thinking. Unlike other cognitive skills, this variable does not appear to follow a completely stable developmental trajectory. While some studies suggest an increase during early childhood (Pasarín-Lavín et al., 2024), others have indicated periods of stagnation or variability in aspects such as originality and fluency (Vong et al., 2020). Additionally, sex differences in divergent thinking expression have also been observed, although findings remain inconsistent across studies. Some studies have reported higher levels in boys (Vong et al., 2020), while others have reported higher levels in girls (Israel-Fishelson et al., 2020). These inconsistencies highlight the complexity of studying divergent thinking during early childhood and reinforce the need for assessment approaches adapted to preschool contexts.

Although several divergent thinking tests exist, most are designed for children aged six years and older, require individual administration, or focus primarily on the final product rather than the creative process. Figurative drawing tasks are widely used in early childhood assessment because they do not require reading or writing skills and allow children to express ideas nonverbally. Accordingly, the present study employed an ad hoc rubric-based drawing task to explore divergent thinking dimensions in children aged 3–5 years within classroom settings.

Teachers and classroom environments may play an important role in fostering divergent thinking during early childhood. Educational practices that encourage exploration, experimentation, and open-ended activities may facilitate children’s expression of creative behaviors in classroom settings (Bibire et al., 2024; Nikkola et al., 2024). In addition, supportive educational environments may help children feel more comfortable expressing ideas and exploring different possibilities during learning activities (Caiman et al., 2021).

Moreover, divergent thinking does not develop in isolation; it interacts with other cognitive skills. Intelligence, understood as a multifaceted capacity associated with reasoning and problem solving (Benedek et al., 2014), has been widely studied in relation to divergent thinking. It is traditionally divided into two types: fluid and crystallized intelligence (Horn & Noll, 1997; Ramírez-Benítez et al., 2016). Fluid intelligence refers to the ability to solve novel problems using complex mental operations, while crystallized intelligence relates to the accumulation of knowledge over time (Krumm et al., 2018). The relationship between intelligence and divergent thinking has been the subject of debate (Plucker et al., 2020), with some studies suggesting that both may contribute to broader cognitive development in early childhood (Kaufman et al., 2013; Silvia, 2015). Previous studies have also reported variability in cognitive and divergent thinking performance according to developmental and sex-related factors, although findings remain inconsistent across studies (Vong et al., 2020).

Pre-literacy skills are another important factor in early cognitive development, as they lay the groundwork for future academic achievement. Pre-literacy development encompasses cognitive and neuromotor skills associated with the early stages of reading and writing acquisition (Jablonski, 2017). This progression has been associated not only with later academic performance but may also play a significant role in socioemotional development (Horowitz-Kraus & Hutton, 2018; Kochhann et al., 2018; Méndez-Freije et al., 2024; Smart et al., 2017). Some authors have also suggested potential associations between divergent thinking and pre-literacy skills, indicating that both constructs may interact during early childhood development (Mourgues et al., 2014).

Further research is needed to better understand how divergent thinking relates to cognitive and pre-literacy skills during early childhood, as well as whether these variables show differences according to sex or school grade. In this context, the present study addressed two main objectives. First, it examined potential differences in divergent thinking and cognitive/pre-literacy skills according to sex and school grade. Second, it explored the associations between divergent thinking dimensions and cognitive and pre-literacy measures in this sample.

Overall, this study aims to provide empirical evidence on sex and age differences in divergent thinking and preliteracy skills in kindergarten populations, as well as to examine the explanatory power of creative variables for preliteracy skills. By analyzing these associations in preschool children, the study seeks to contribute to the current literature on creativity and early childhood development within educational contexts (Harju-Luukkainen et al., 2022).

## 2. Materials and Methods

### 2.1. Participants

This study used a non-random convenience sample of 41 boys (51.25%) and 39 girls (48.75%), aged between 3 and 5 years (*M* = 4.44; *SD* = 0.57) from three urban preschools in the Principality of Asturias (Spain). Participants were between 47 and 71 months old (*M* = 58.60; *SD* = 6.63), equivalent to a mean age of 4.44 years (*SD* = 0.57). The sample was split into two groups (Table 1) according to school year: 2nd year of Preschool Education (*n* = 45; 56.25%); 3rd year of Preschool Education (*n* = 35; 43.75%). All children came from middle socioeconomic status families.

All participating schools were located in urban areas with predominantly middle socioeconomic backgrounds, and no schools serving highly disadvantaged populations were included. Therefore, the sample can be considered relatively homogeneous in terms of socioeconomic status.

No missing data were identified in the dataset. However, because three participants were below the recommended age range for the AEI-R, mean substitution was applied to these cases exclusively for analyses requiring complete datasets.

According to Tabachnick and Fidell (2013), the recommended sample size for multiple regression analyses is *N* ≥ 50 + 8*m* (where *m* is the number of predictors). Therefore, the sample size was considered acceptable for regression analyses.

### 2.2. Instruments

A simple test was designed ad hoc to assess divergent thinking in the sample, given the lack of tools adapted to this stage and the complexity of the construct.

It was inspired by four figurative tests for the school population: the Torrance Test of Creative Thinking (TTCT; Torrance, 1966), the Test of Creative Imagination (PIC; Artola et al., 2008), the Test of Children’s Creativity (TCI; Romo et al., 2016) and the Creative Intelligence Test (CREA; Corbalán et al., 2003).

The rubric’s construct validity relies on three established creativity assessments. First, the four core dimensions—fluency, flexibility, originality, and elaboration—are grounded in Torrance’s (1966) classic divergent thinking framework. Within this model, fluency quantifies the generated responses or graphic elements; flexibility captures response diversity; originality reflects the infrequency of ideas; and elaboration measures the degree of detail and refinement. Second, the PIC Test (Artola et al., 2008) informed the organization of observable creativity indicators and structured scoring criteria. Third, the CREA test (Corbalán et al., 2003) reinforced the assessment’s focus on idea generation and divergent production.

Procedurally, the instrument adapts the framework of the TCI test (Romo et al., 2016), which evaluates creativity through a figurative task capturing both process and product. The TCI features an initial formulation phase followed by a drawing phase, assessing both the final output and the underlying creative decisions. Mirroring this approach, the developed rubric differentiates between process indicators (the child’s behavior during execution) and product indicators (the characteristics of the final drawing). Specifically, four TCI scoring principles guided the rubric’s design: (1) atypical material transformation inspired indicators for material exploration, unconventional tool use, and variation in textures, colors, and shapes; (2) interaction among drawing elements informed indicators for composition and organization; (3) the TCI’s verbal integration led to the inclusion of title and explanation metrics; and (4) the concepts of invented elements and distancing from the initial model informed the indicators for original combinations, flexibility, and narrative enrichment.

To establish content validity, the expert panel evaluated the rubric items and scoring criteria, while inter-rater agreement analyses verified the alignment of items with their respective divergent thinking dimensions. Fleiss’ Kappa coefficients yielded κ = 0.84 for the process rubric and κ = 0.83 for the product rubric, demonstrating strong consensus among the three judges. Following this expert consensus, a qualitative pre-testing phase was conducted via the individual administration of the task to three preschool children. This step functioned strictly as a cognitive and operational debriefing to verify task comprehension, instructions clarity, and administrative feasibility under real conditions, rather than a statistical pilot study. Although these initial steps provide rigorous evidence of content validity and procedural feasibility, broader psychometric evaluations are required to confirm the instrument’s structural properties in larger samples. To support open science and facilitate replication, the complete rubric, scoring criteria, task materials, and illustrated scoring examples are available in the Appendix A.

The final version of the test adopts a multidimensional approach (Lansing-Stoeffler & Daley, 2023) that combines analysis of the divergent thinking process (Zeng et al., 2011) and the divergent thinking product (Fairweather & Cramond, 2012), based on the four variables that define divergent thinking. Figurative drawing is proposed because of its ability to capture children’s imaginations and minimize the influence of cultural factors (Pisotska, 2022). This type of test has been shown to be effective in assessing divergent thinking in young children, particularly in familiar settings such as the classroom, ensuring greater ecological validity (Long & Wang, 2022). In this sense, the designed rubric consists of 35 items in two sections: divergent thinking process and divergent thinking product, each subdivided into four sub-sections: fluency, flexibility, originality, and elaboration. Thus, process indicators refer to behavioral and strategic aspects of creativity, whereas product indicators refer to the quantity, diversity, originality, and elaboration of the final graphic production.

The test is administered in groups of 4 students, with a total time of approximately 30 min, including application instructions and exploration of materials. The scores for each item range on a Likert scale from 1 to 4 (1 = never; 2 = sometimes; 3 = often; 4 = always), resulting in a mean score for each sub-variable (fluency in process, flexibility in process, originality in process, elaboration in process, fluency in product, flexibility in product, originality in product and elaboration in product). The Likert scale did not rely on a general subjective impression, but on structured scoring criteria defined in the rubric, based on observable indicators such as number of ideas, use of materials, number of changes, originality of combinations, and level of detail in the drawing. Therefore, scoring was based on specific behavioral and product-based indicators rather than global subjective judgments. This rubric-based approach allowed for a more comprehensive assessment of divergent thinking by considering both the creative process and the final creative product.

Overall divergent thinking scores were calculated by averaging the scores obtained in the process and product dimensions for each divergent thinking variable (fluency, flexibility, originality, and elaboration), resulting in overall fluency, flexibility, originality, and elaboration scores used in the regression analyses.

After administering the test and completing the rubric for all participants, high reliability was determined, with Cronbach’s Alpha coefficient = 0.89. All drawings and process observations were scored by a trained researcher following detailed scoring criteria defined in the rubric. The scoring procedure was based on observable indicators and specific criteria for each dimension to reduce subjectivity and ensure consistency in the evaluation process. Reliability analyses were also examined across the divergent thinking dimensions to ensure consistency in the scoring process. Clear scoring guidelines were established to reduce subjectivity and ensure consistency in the evaluation process. All tasks were administered in Spanish, the participants’ mother tongue, in quiet and familiar classroom settings.

To assess intelligence and pre-literacy development, the Aptitudes in Early Childhood Education—Revised test (TEA Ediciones R&D Department, 2018) was administered. This is a tool designed to measure a variety of intellectual skills and pre-literacy development in students aged 4 to 6 years, who are at the end of the Early Childhood Education stage (2nd and 3rd year). It consists of 77 items distributed in different areas: verbal aptitude, quantitative aptitude, spatial orientation, auditory memory, and visuo-motor skills to establish overall intellectual aptitude and pre-literacy development. The test can be administered individually or in a group, with a total time of approximately 40 min, including application instructions. The scores in each aptitude area are summed to obtain a total score in intellectual skills, which is then converted into a typical and centile score. In addition, pre-literacy development is determined by summing direct scores in skills related to verbalization, spatial orientation, auditory memory and visuo-motor skills. The AEI-R was developed with the participation of a large sample of 12,440 Spanish students, thus ensuring high levels of reliability, with a Cronbach’s Alpha = 0.87.

### 2.3. Procedure

The study was approved by an Ethics Committee (CEISH-UPV/EHU, BOPV 32-2022; approved in May 2022) and conducted in accordance with the ethical principles outlined in the Declaration of Helsinki (World Medical Association, 2013). All procedures complied with current institutional and national regulations governing research involving human participants. In this sense, written informed consent was obtained from all children’s legal guardians prior to participation. In addition, prior to the assessment sessions, the children received an age-appropriate oral explanation of the activities and were asked whether they wished to participate. Participation was voluntary, and children were informed that they could stop the activities at any time if they did not wish to continue.

The assessments were administered within the school timetable, in quiet and familiar classroom environments, by a trained researcher from the University of Oviedo. Testing sessions were organized in small groups of four students to accommodate the children’s developmental needs and the characteristics of the tasks. Data collection was completed over a two-week period, with no more than three days between sessions, to minimize fatigue and maintain consistent testing conditions.

All tasks and instructions were administered in Spanish, the participants’ mother tongue, to facilitate comprehension and reduce potential language-related difficulties during assessment. The administration procedures were adapted to the developmental characteristics of preschool children, including flexible pacing, simplified instructions when necessary, and the use of familiar classroom materials and environments to promote engagement and reduce situational anxiety.

Given that the AEI-R is standardized for children aged 4 years and older, three participants in the sample were below the recommended age range for normative interpretation. In these cases, AEI-R scores were replaced by the sample mean exclusively for analyses requiring complete datasets, to avoid case exclusion due to the relatively small sample size. Given the limited number of affected cases, the potential impact on the analyses was considered minimal and results were interpreted with caution.

### 2.4. Statistical Analysis

Data analyses were conducted using IBM SPSS Statistics 26.0. Skewness and kurtosis coefficients were examined to assess normality, and Levene’s test was used to verify homoscedasticity. Following Kline’s (2013) recommendations, skewness values below 2 and kurtosis values below 7 were considered indicative of acceptable normality for the use of parametric analyses. Pearson’s correlation analyses were also calculated to explore associations among the variables analyzed in this study. Given the high correlations observed among some divergent thinking dimensions, multicollinearity diagnostics were subsequently examined in the regression analyses to evaluate potential overlap among the study variables. Subsequently, multivariate analyses of covariance (MANCOVA) were conducted, controlling for participants’ age in months. Follow-up univariate analyses of covariance (ANCOVA) were examined for each dependent variable when appropriate. To reduce the risk of Type I error associated with multiple comparisons, Bonferroni correction was applied to the univariate analyses, resulting in an adjusted significance threshold of *p* < .006. Statistical significance was set at *p* ≤ .05 for all two-tailed tests.

Effect sizes for the MANCOVA and ANCOVA analyses were calculated using partial eta squared (ηp^2^). Values of 0.01, 0.06, and 0.14 were considered small, medium, and large effects, respectively. For the regression analyses, R^2^, adjusted R^2^, ΔR^2^, and ANOVA F statistics were examined to evaluate model fit. Effect sizes for the regression analyses were interpreted using Cohen’s f^2^ (Cohen, 1988), where values of 0.02, 0.15, and 0.35 represent small, medium, and large effects, respectively. Given the exploratory nature of the study, effect sizes were interpreted cautiously and considered complementary to statistical significance.

A Hierarchical linear regression analysis was conducted using the enter method. Sex was entered in Step 1 as a control variable because previous multivariate analyses revealed differences according to sex in some divergent thinking variables. In addition, considering the relatively small sample size, a parsimonious modeling strategy was adopted to reduce model complexity and avoid overfitting. In Step 2, the divergent thinking dimensions (fluency, flexibility, originality, and elaboration) were simultaneously entered into the models to examine their statistical associations with the cognitive and pre-literacy variables analyzed in the study. Prior to the analyses, multicollinearity diagnostics were examined. Tolerance and variance inflation factor (VIF) values were within acceptable ranges (tolerance = 0.192–0.850; VIF = 1.177–5.210), indicating that multicollinearity was not a serious concern despite the correlations among some divergent thinking variables. Given the cross-sectional design of the study, regression analyses were interpreted as statistical associations rather than evidence of longitudinal or causal relationships.

## 3. Results

This section presents the descriptive, correlational and inferential results obtained from the analyses conducted on the preschool sample.

### 3.1. Descriptive Statistics

Table 2 presents the descriptive statistics for all study variables derived from the Divergent Thinking Drawing Test and the AEI-R. According to Kline’s (2013) criteria (skewness values below 2 and kurtosis below 7), the skewness and kurtosis values obtained were considered acceptable for the use of parametric analyses and were applied in subsequent stages.

### 3.2. Correlational Analyses

Pearson correlation analyses were conducted to explore associations between divergent thinking, cognitive, and pre-literacy variables (Table 3). Several significant mmi were found among the dimensions of the Divergent Thinking Drawing Test. Likewise, significant correlations were found among most AEI-R variables.

Several significant correlations were also observed between divergent thinking and cognitive/pre-literacy variables. Fluency (process and product) showed positive correlations with auditory memory, whereas process elaboration was positively correlated with verbal aptitude, visuo-motor skills, and pre-literacy development. Product flexibility was positively associated with auditory memory and visuo-motor skills, and product elaboration showed positive correlations with verbal aptitude and pre-literacy development.

### 3.3. Multivariate Analyses

To examine group differences according to sex and school year, four multivariate analyses of covariance (MANCOVA) were conducted, including age a covariate. Follow-up univariate analyses of covariance (ANCOVA) were subsequently examined for each dependent variable. To reduce the risk of Type I error associated with multiple comparisons, Bonferroni correction was applied. The results are presented below.

#### 3.3.1. Sex and Grade-Level Differences in Divergent Thinking Variables

Firstly, a MANCOVA was conducted to examine differences in divergent thinking variables according to sex while controlling for age. The multivariate effect of sex was statistically significant, Wilks’ λ = 0.761, *F*(8,70) = 2.747, *p* = .011, ηp^2^ = 0.239. Follow-up ANCOVAs revealed differences in process and product elaboration, respectively, at the conventional significance level (*p* < .05), with boys obtaining slightly higher scores than girls in both variables: process elaboration, *F*(1,77) = 4.443, *p* = .038, ηp^2^ = 0.055; and product elaboration, *F*(1,77) = 4.701, *p* = .033, ηp^2^ = 0.058. However, these effects did not remain statistically significant after applying the adjusted significance threshold (*p* < .006). No statistically significant differences were found for the remaining divergent thinking variables.

Secondly, a second MANCOVA was performed to examine differences in divergent thinking variables according to school year (grade level), while controlling for age. The results indicated no statistically significant differences in divergent thinking variables as a function of school year, Wilks’ λ = 0.863, *F*(8,70) = 1.387, *p* = .218, ηp^2^ = 0.137.

#### 3.3.2. Sex and Grade-Level Differences in Cognitive and Pre-Literacy Skills

Two MANCOVAs were conducted to examine differences in cognitive and pre-literacy skills according to sex and grade level, while controlling for age.

Regarding sex differences, the MANCOVA results indicated no statistically significant differences in cognitive and pre-literacy skills, Wilks’ λ = 0.928, *F*(6,72) = 0.935, *p* = .475, ηp^2^ = 0.072.

Similarly, when examining differences in cognitive and pre-literacy variables according to school year (grade level), the MANCOVA results also revealed no statistically significant differences, Wilks’ λ = 0.906, *F*(6,72) = 1.246, *p* = .293, ηp^2^ = 0.094.

### 3.4. Explanatory Power of Creative Variables on Preliteracy Skills

A hierarchical linear regression analysis was conducted to examine the explanatory power of divergent thinking dimensions (fluency, flexibility, originality, and elaboration) over pre-literacy skills, measured through the Preliteracy Development Index of the AEIR test. Sex variable was entered in Step 1 as a control variable, given that prior multivariate analyses indicated significant sex-based differences across several divergent thinking metrics. In Step 2, the four divergent thinking dimensions were entered simultaneously using the forced entry method to evaluate their unique incremental contribution to pre-literacy development. Model fit was evaluated using R^2^, adjusted R^2^, ΔR^2^, and ANOVA *F* statistics.

Multicollinearity diagnostics were evaluated using tolerance and variance inflation factor (VIF) values. Tolerance values ranged from 0.19 to 0.85, while VIF values ranged from 1.18 to 5.21. Although these values indicate relatively elevated associations among specific divergent thinking dimensions, they remain within acceptable psychometric thresholds.

As shown in Table 4, the regression model for pre-literacy development became statistically significant when the creativity variables were included in Step 2, explaining a total of 14.7% of the variance. Moreover, within this regression model, creative elaboration emerged as the sole creative variable with significant explanatory power over pre-literacy development.

## 4. Discussion

The main aim of the present study was to examine the explanatory power of divergent thinking variables over cognitive skill and pre-literacy skills in preschool children aged 3 to 5 years old. Overall, these findings contribute to a clearer understanding of the relationship between divergent thinking and cognitive and pre-literacy development in early childhood and may help to inform and guide future research in this area.

This work represents an initial step toward establishing a creativity assessment framework for this developmental stage, aligned with the critical needs highlighted by Rapti and Sapounidis (2024) and Nikkola et al. (2024). Although most research on divergent thinking has focused on older populations, empirical studies involving early childhood and kindergarten samples remain scarce (Massy et al., 2025). In this context, rather than providing definitive conclusions, the value of the present study lies in establishing a foundational baseline that opens avenues for broader future research. Given that early childhood represents a critical period in human development, further evidence supporting an explanatory link between creative dimensions and foundational skills would underscore the importance of integrating divergent thinking more systematically into early education policy and practice from the outset of schooling.

Considering performance patterns, participants scored higher in fluency and flexibility, while their scores in originality and elaboration were lower. This pattern is consistent with previous studies highlighting the developmental characteristics of creative expression at this age (Sawyer, 2012). In fact, these findings may suggest the educational relevance of activities that encourage not only idea generation, but also the progressive enrichment and refinement of ideas.

Regarding sex differences, boys obtained significantly higher scores than girls in creative elaboration. However, previous research has reported inconsistent findings concerning sex differences in divergent thinking, with some studies indicating higher scores in girls and others in boys (Israel-Fishelson et al., 2020; Vong et al., 2020). These findings suggest that creativity does not consistently manifest at higher levels in either boys or girls, but rather may vary according to contextual, cultural, and educational factors. The present findings are consistent with the need for caution when interpreting sex-related differences in divergent thinking during early childhood, especially in relatively small samples.

With respect to school grade, the present results do not provide evidence of grade-level differences in divergent thinking in this sample. This finding is consistent with previous research suggesting that the development of divergent thinking during early childhood does not always follow a linear trajectory and may show considerable variability across educational stages (Alfonso-Benlliure & Romo-Santos, 2016). Moreover, divergent thinking in early childhood may be strongly influenced by contextual and educational factors, such as classroom environment and opportunities for creative expression, which could attenuate differences associated with school grade alone (Duval et al., 2022).

For its part, when cognitive and pre-literacy skills were analysed, no statistically significant differences were found according to sex or school grade after applying the adjusted significance threshold. These results may suggest that, similarly to creativity, cognitive and pre-literacy skills in early childhood do not necessarily develop in a strictly linear manner, but instead may follow heterogeneous developmental trajectories shaped by a range of contextual and maturational factors (Larsen & Erbeli, 2026).

On the other hand, in relation to the main aim of this study, which was to analyze the explanatory power of creative skills for preliteracy abilities, the hierarchical regression analysis revealed that creative elaboration was the only variable showing significant explanatory power for preliteracy skills. These findings are consistent with previous research suggesting that creative elaboration involves the activation of higher-order cognitive processes associated with general cognitive functioning, such as planning, organization, attention to detail, among others (Pasarín-Lavín et al., 2024). Future research should further examine the conceptual boundaries between elaboration and cognitive development to determine whether elaboration represents a distinct creative construct, a manifestation of broader cognitive ability, or a combination of both. This is particularly relevant given the moderate-to-high correlations observed among some divergent thinking dimensions, which may reflect partial conceptual overlap between rubric dimensions.

Furthermore, significant correlations emerged between divergent thinking, cognitive skills and pre-literacy development, suggesting associations between these constructs at the preschool stage. These findings are consistent with studies that highlight the interdependence between divergent thinking and intelligence and preliteracy skills (Kaufman et al., 2013; Silvia, 2015). However, the observed associations were generally modest and should not be interpreted as evidence of causal or developmental effects. From an educational perspective, these findings may suggest a trend supporting the inclusion of open-ended activities, drawing tasks, storytelling, and exploratory classroom experiences as valuable contexts for both observing and fostering creative expression (Rawlings et al., 2025).

Despite the contributions of this study, several limitations should be acknowledged. First, the non-randomized sampling procedure and modest sample size constrain the generalizability of the findings. Future research would benefit from larger and more representative samples, which could yield more robust and widely generalizable results. The use of a convenience sample from a limited geographic area and predominantly middle socioeconomic status families may restrict the generalizability of the findings.

Second, although Bonferroni correction was applied to reduce the risk of Type I error in the univariate analyses, the number of statistical tests conducted requires caution when interpreting significant findings.

Third, the divergent thinking assessment was based on an ad hoc instrument derived from widely used tests designed for older populations. Although internal consistency and inter-rater agreement for item classification were examined, further research is needed to provide stronger evidence of its validity. Therefore, given the characteristics of the present sample, the findings should be interpreted as preliminary associations rather than evidence of developmental change, predictive validity, or screening utility.

Another aspect to consider is the absence of longitudinal analyses that allow us to observe how these variables develop over time. Divergent thinking, cognitive skills and pre-literacy development are dynamic constructs that can be influenced by various contextual and educational factors. Therefore, it would be useful to carry out longitudinal studies that provide a developmental perspective and allow for the identification of developmental patterns across early childhood. Such designs would also be necessary to determine whether divergent thinking dimensions are related to later cognitive or literacy outcomes.

In addition, incorporating students with Special Educational Needs (SEN) in future research would enhance our understanding of how these constructs interact within heterogeneous educational settings. Including participants with diverse developmental profiles would promote a more inclusive and holistic perspective on the relationships among divergent thinking, intelligence, and pre-literacy development, providing practical guidance for addressing diversity in the early years. Future studies should also consider broader socioeconomic and educational contexts to examine whether the present findings are replicated in more heterogeneous samples.

In conclusion, these findings underscore the importance of examining the interrelationships among divergent thinking, intelligence, and pre-literacy skills during the preschool years. The results suggest that some divergent thinking dimensions, particularly elaboration, present significant explanatory power with pre-literacy variables in this sample. However, these associations should be interpreted cautiously due to the modest sample size and the preliminary nature of the rubric-based assessment. By addressing current methodological limitations and adopting broader, longitudinal perspectives, future research may contribute to a more nuanced understanding of how creative and cognitive processes are related during early development. Future studies should examine whether elaboration remains associated with cognitive and literacy-related skills over time and whether structured educational experiences can support both creative expression and early learning in preschool contexts.

## Figures and Tables

**Table 1:** Sample Demographic Characteristics.

Groups	Sex			Age (Months)	
	N	Girls	Boys	*M*	*SD*
2nd grade	45	23	22	52.8	3.99
3rd grade	35	16	19	64.8	3.16
Total	80	39	41	58.6	6.63

Note. *M* = Mean; *SD* = Standard deviation; Age is reported in months.

**Table 2:** Descriptive Statistics of Study Variables provided by the Divergent Thinking Drawing Test and the AEI-R.

**Divergent Thinking Drawing Test Variables**	Descriptive Statistics			
	*M*	*SD*	Skewness	Kurtosis
**Process**				
Fluency	2.41	0.67	0.451	−0.208
Flexibility	2.13	0.61	0.646	0.320
Originality	1.66	0.72	0.987	−0.268
Elaboration	1.95	0.76	0.765	−0.050
**Product**				
Fluency	2.79	0.56	−0.401	−0.058
Flexibility	2.35	0.56	0.067	−0.056
Originality	1.47	0.54	1.430	1.763
Elaboration	1.51	0.58	1.926	3.591
**AEI-R test variables**	Descriptive Statistics			
	*M*	*SD*	Skewness	Kurtosis
Verbal	49.04	11.21	0.073	−0.808
Quantitative	45.24	9.37	0.027	−0.292
Spatial Orientation	44.00	9.63	1.052	1.414
Auditory memory	47.72	11.14	0.875	0.399
Visuomotor	43.15	8.51	0.279	−0.285
Pre-Literacy Development	42.19	8.96	0.321	−0.542

Note. *M = Mean; SD = Standard deviation. Variable names and category labels are presented in bold.*

**Table 3:** Pearson Correlations for all Study Variables.

	1	2	3	4	5	6	7	8	9	10	11	12	13	14
1. FLUpc	–													
2. FLEpc	0.80 ***	–												
3. ORpc	0.43 ***	0.63 ***	–											
4. ELApc	0.36 **	0.39 ***	0.52 ***	–										
5. FLUpd	0.44 ***	0.59 ***	0.45 ***	0.57 ***	–									
6. FLEpd	0.34 **	0.58 ***	0.56 ***	0.54 ***	0.86 ***	–								
7. ORpd	0.42 ***	0.63 ***	0.86 ***	0.56 ***	0.52 ***	0.60 ***	–							
8. ELApd	0.35 **	0.42 ***	0.39 ***	0.68 ***	0.54 ***	0.57 ***	0.54 ***	–						
9. V	−0.09	−0.09	0.01	0.29 *	0.18	0.18	0.05	0.23 *	–					
10. Q	0.02	−0.04	0.01	0.14	0.07	0.02	−0.05	0.06	0.60 ***	–				
11. SO	−0.08	−0.17	−0.14	0.21	0.03	−0.04	−0.15	0.13	0.40 ***	0.46 ***	–			
12. AM	0.23 *	0.18	0.20	0.17	0.25 *	0.24 *	0.12	0.14	0.14	0.18	0.19	–		
13. VM	−0.09	0.00	0.06	0.31 **	0.21	0.24 *	0.08	0.22	0.42 ***	0.38 ***	0.46 ***	0.17	–	
14. PD	−0.05	−0.09	−0.2	0.30 **	0.18	0.17	−0.01	0.24 *	0.66 ***	0.55 ***	0.78 ***	0.34 **	0.81 ***	–

Note. FLUpc = Fluency in the process; FLEpc = Flexibility in the process; ORpc = Originality in the process; ELApc = Elaboration in the process; FLUpd = Fluency in the product; FLEpd = Flexibility in the product; ORpd = Originality in the product; ELApd = Elaboration in the product; V = Verbal aptitude; Q = Quantitative aptitude; SO = Spatial orientation; AM = Auditory memory; VM = Visuomotor; PD = Pre-Literacy development. Significant correlations between divergent thinking and AEI-R variables are presented in bold. * *p* < .05; ** *p* < .01; *** *p* < .001.

**Table 4:** Hierarchical Regression Analysis.

Regression Models	Pre-Literacy Development
Step 1	
Constant	41.390 (1.402 ***)
Sex	0.092 (0.814)
R^2^ = 0.008	
*F*(1, 78) = 0.663	
*p* = .418	
Step 2	
Constant	39.453 (4.999 ***)
Sex	−0.040 (−0.345)
Fluency	−0.096 (−0.449)
Flexibility	0.017 (0.069)
Originality	−0.265 (−1.587)
Elaboration	0.506 (3.394 ***)
R^2^ = 0.147	
ΔR^2^ = 0.139	
*F*(5, 74) = 2.561 *	
*p* = .034	

Note. Values in the table are standardized regression coefficients (β); values in parentheses correspond to *t* statistics. R^2^ = variance explained; ΔR^2^ = change in explained variance between Step 1 and Step 2. * *p* < .05; *** *p* < .001.

## Data Availability

The datasets generated and analyzed during the current study are not publicly available due to ethical and privacy restrictions involving child participants.

## Abbreviations

The following abbreviations are used in this manuscript:

AEI-R	Aptitudes in Early Childhood Education—Revised
FLUpc, FLEpc, ORpc, ELApc	Fluency, Flexibility, Originality, Elaboration—process
FLUpd, FLEpd, ORpd, ELApd	Fluency, Flexibility, Originality, Elaboration—product
V	Verbal aptitude
C	Quantitative aptitude
OE	Spatial orientation
MA	Auditory memory
VM	Visuo-motor skills
ML	Pre-literacy development
AI	Overall intellectual aptitude
SEN	Special Educational Needs
FICYT	Foundation for the Promotion of Applied Scientific Research and Technology in Asturias

## Appendix A

Table A1: Process rubric; Table A2: Product rubric; Table A3: Operational definitions of DT dimensions; Table A4: Scoring criteria for divergent thinking dimensions; Table A5: Creative drawing task instructions and materials. These materials are provided to facilitate replication and use of the instrument in future research and educational contexts.

**Table A1:** Process rubric used to assess behavioral indicators of divergent thinking during task execution.

Dimension	Indicator	1 (Low)	2	3	4 (High)
Fluency	Facial and body expression	Very limited expression and involvement	Occasional expression and involvement	Frequent expression and involvement	Very expressive and actively engaged throughout the task
	Verbal interaction and curiosity	Rarely communicates or asks questions	Occasionally communicates	Frequently communicates and shows curiosity	Constant verbal interaction and curiosity
	Non-verbal communication	Minimal gestures or non-verbal interaction	Some non-verbal communication	Frequent non-verbal communication	Very active non-verbal communication and interaction
	Exploration and use of materials	Uses very few materials	Uses some materials	Uses several materials	Uses a wide variety of materials
Flexibility	Variety of facial and body expression	Shows little variation	Some variation	Frequent variation	Very diverse expressions and behaviors
	Variety of verbal interaction and curiosity	Limited variety in communication	Some variety	Frequent variety	Highly varied communication and curiosity
	Variety of non-verbal communication	Limited non-verbal variation	Some variation	Frequent variation	Highly varied non-verbal communication
	Variety of materials used	Uses same materials repeatedly	Uses some different materials	Uses several different materials	Uses many different materials
	Variety of textures, colors, and shapes	Little variation	Some variation	Frequent variation	High diversity of textures, colors, and shapes
Originality	Unusual use of materials	Very conventional use	Occasionally unusual	Frequently unusual	Highly original use of materials
	Unusual use of textures, colors, and shapes	Very conventional combinations	Some unusual combinations	Frequent unusual combinations	Highly original combinations
	Originality of title	Very common title	Some originality	Original title	Highly original and creative title
	Originality in explanation of drawing	Very simple explanation	Some original elements	Original explanation	Highly original and imaginative explanation
Elaboration	Care and detail in use of materials	Very little detail	Some detail	Detailed work	Very detailed and careful work
	Care and detail in textures, colors, and shapes	Very little detail	Some detail	Detailed	Highly detailed and refined
	Detail and relevance of title	Very simple title	Some detail	Detailed title	Very detailed and meaningful title
	Detail and explanation of drawing	Very brief explanation	Some explanation	Detailed explanation	Very detailed and elaborate explanation

Note. All indicators were scored on a four-point Likert scale based on observable behaviors during task execution. Higher scores indicate greater presence of divergent thinking behaviors during the creative process.

**Table A2:** Product rubric used to assess divergent thinking based on the characteristics of the final drawing.

Dimension	Indicator	1 (Low)	2	3	4 (High)
Fluency	Number of materials used	Uses very few materials	Uses some materials	Uses several materials	Uses many different materials
	Number of textures	Very few textures	Some textures	Several textures	Many textures
	Number of colors	Uses very few colors	Uses some colors	Uses several colors	Uses many colors
	Number of shapes	Uses very few shapes	Uses some shapes	Uses several shapes	Uses many shapes
Flexibility	Diversity of materials	Very low diversity	Some diversity	High diversity	Very high diversity
	Diversity of textures	Very low diversity	Some diversity	High diversity	Very high diversity
	Diversity of colors	Very low diversity	Some diversity	High diversity	Very high diversity
	Diversity of shapes	Very low diversity	Some diversity	High diversity	Very high diversity
Originality	Unusual use of materials	Very conventional use	Occasionally unusual	Frequently unusual	Highly original use
	Unusual use of textures	Very conventional	Some unusual combinations	Frequent unusual combinations	Highly original combinations
	Unusual use of colors	Very conventional color use	Some unusual combinations	Frequent unusual combinations	Highly original color combinations
	Unusual use of shapes	Very conventional shapes	Some unusual shapes	Frequent unusual shapes	Highly original shapes
	Special creative details (expansion, rotation, connections, shading, background)	No special details	One special detail	Several special details	Many creative details integrated
Elaboration	Visual composition complexity	Very simple composition	Some complexity	Complex composition	Very complex and well-structured composition
	Enrichment of drawing (shading, perspective, texture, etc.)	No enrichment	Some enrichment	Considerable enrichment	Highly enriched drawing
	Expressiveness of drawing	Very little expressiveness	Some expressiveness	Expressive drawing	Highly expressive and detailed drawing

Note. Product indicators were scored based on the characteristics of the final drawing. Higher scores indicate greater fluency, flexibility, originality, and elaboration in the creative product. The “special creative details” indicator was included to capture qualitative aspects of creativity such as expansion of the drawing space, rotation of figures, connection of shapes, use of shading, and inclusion of background elements.

**Table A3:** Operational definition of divergent thinking dimensions used in the scoring process.

Dimension	Indicator
Fluency	Number of ideas, elements, materials, or actions generated during the task
Flexibility	Variety and diversity of ideas, materials, textures, colors, shapes, or strategies used
Originality	Unusual, infrequent, or novel use of materials, combinations, titles, or explanations
Elaboration	Level of detail, complexity, refinement, and enrichment of the drawing and explanation

Note. These operational definitions were used to develop the scoring rubrics and guide the evaluation of both the creative process and the final product. Scores were assigned based on observable indicators corresponding to each divergent thinking dimension.

**Table A4:** Scoring criteria used to rate divergent thinking indicators on the four-point Likert scale.

Dimension	Fluency	Flexibility	Originality	Elaboration
1	Very few ideas, elements, or actions	Very little variety in materials, strategies, or elements	Very conventional responses	Very simple work with little detail
2	Some ideas, elements, or actions	Some variety in materials, strategies, or elements	Occasionally unusual responses	Some details or development
3	Several ideas, elements, or actions	Considerable variety in materials, strategies, or elements	Frequently unusual or novel responses	Detailed and developed work
4	Many ideas, elements, or actions	High diversity of materials, strategies, or elements	Highly original and novel responses	Highly detailed, complex, and refined work

Note. These scoring criteria were applied to both process-based and product-based indicators of divergent thinking. Scores were assigned based on observable behaviors during task execution and characteristics of the final drawing. Higher scores indicate greater presence of divergent thinking abilities.

**Table A5:** Creative drawing task instructions and administration procedure.

Setting	− Familiar classroom environment − Spacious and well-lit room − Small groups of four children
Materials Provided	− Paper sheets − Colored cardstock − Colored pencils and crayons − Markers − Scissors and glue − Glitter and collage materials − Eraser and sharpener
Instructions Given to Children	“You are going to create your own drawing using these materials. You can create anything you want. Try to create something original and different. You can use as many materials as you like, combine them, and change your idea if you want. At the end, you will give a title and explain your drawing.”
Task Duration	− Approximately 30 min
Observation Procedure	− Behavioral indicators recorded using the process rubric − Title and explanation requested at the end of the activity

Note. Administration procedure and instructions for the creative drawing task used to assess divergent thinking. The table summarizes the task setting, materials provided, instructions given to children, duration of the activity, and observation and scoring procedure.
